# FcγRIIB potentiates differentiation of myeloid-derived suppressor cells to mediate tumor immunoescape

**DOI:** 10.7150/thno.66575

**Published:** 2022-01-01

**Authors:** Lei Wu, Yanquan Xu, Huakan Zhao, Yu Zhou, Yu Chen, Shuai Yang, Juan Lei, Jiangang Zhang, Jingchun Wang, Yongzhong Wu, Yongsheng Li

**Affiliations:** 1 Department of Medical Oncology, Chongqing University Cancer Hospital, Chongqing 400030, China.; 2 Chongqing Key Laboratory for Intelligent Oncology in Breast Cancer, Chongqing University Cancer Hospital, Chongqing 400030, China.; 3 Clinical Medicine Research Center, Xinqiao Hospital, Army Medical University, Chongqing 400037, China.; 4 Department of Radiotherapy, Chongqing University Cancer Hospital, Chongqing 400030, China.

**Keywords:** myeloid-derived suppressor cells, Fc gamma receptor IIB, tumor microenvironment, granulocyte-macrophage colony stimulating factor, immunosuppression, anti-tumor therapy, Sp1 signaling

## Abstract

**Background:** FcγRIIB, the sole inhibitory receptor of the Fc gamma receptor family, plays pivotal roles in innate and adaptive immune responses. However, the expression and function of FcγRIIB in myeloid-derived suppressor cells (MDSCs) remains unknown. This study aimed to investigate whether and how FcγRIIB regulates the immunosuppressive activity of MDSCs during cancer development.

**Methods:** The MC38 and B16-F10 tumor-bearing mouse models were established to investigate the role of FcγRIIB during tumor progression. FcγRIIB-deficient mice, adoptive cell transfer, mRNA-sequencing and flow cytometry analysis were used to assess the role of FcγRIIB on immunosuppressive activity and differentiation of MDSCs.

**Results:** Here we show that FcγRIIB was upregulated in tumor-infiltrated MDSCs. FcγRIIB-deficient mice showed decreased accumulation of MDSCs in the tumor microenvironment (TME) compared with wild-type mice. FcγRIIB was required for the differentiation and immunosuppressive activity of MDSCs. Mechanistically, tumor cell-derived granulocyte-macrophage colony stimulating factor (GM-CSF) increased the expression of FcγRIIB on hematopoietic progenitor cells (HPCs) by activating specificity protein 1 (Sp1), subsequently FcγRIIB promoted the generation of MDSCs from HPCs *via* Stat3 signaling. Furthermore, blockade of Sp1 dampened MDSC differentiation and infiltration in the TME and enhanced the anti-tumor therapeutic efficacy of gemcitabine.

**Conclusion:** These results uncover an unrecognized regulatory role of the FcγRIIB in abnormal differentiation of MDSCs during cancer development and suggest a potential therapeutic target for anti-tumor therapy.

## Introduction

Tumor progression is accompanied by infiltration of a large number of immune cells in the tumor microenvironment (TME) 1. The immunosuppressive TME plays a critical role in determining the outcome of tumor progression or remission 2. Of the infiltrating immune cells in the TME, myeloid-derived suppressor cells (MDSCs) are the predominant population 3. Studies suggest that the frequency of MDSC is associated with tumor stage, burden and metastasis, while a massive accumulation of circulating MDSCs correlates with poor prognosis in cancer patients 4, 5. MDSCs are a heterogeneous group of immature hematopoietic cells that originate from multipotent hematopoietic progenitor cells (HPCs) and are recruited to the TME by tumor-secreted and host-secreted molecules, such as granulocyte-macrophage colony stimulating factor (GM-CSF) 6. MDSCs are characterized by a CD11b^+^Gr1^+^ phenotype, and can be further classified into monocytic (mMDSC, CD11b^+^Ly6C^high^Ly6G^-^) and granulocytic (gMDSC, CD11b^+^Ly6G^+^Ly6C^low^) subpopulations in tumor-bearing mice 7. Both of these subpopulations can suppress the activity of antigen-activated CD8^+^ T cells through multiple mechanisms. MDSCs express arginase and inducible nitric oxide synthase (iNOS), thus depriving arginine in the TME and suppressing the proliferation of T cells 8. They also highly express programmed death-ligand 1 (PD-L1) and reactive oxygen species (ROS) to suppress the activation of cytotoxic T lymphocytes (CTLs) 9. Additionally, MDSCs secrete prostaglandin E_2_ (PGE_2_), calcium-binding protein S100A8/A9, transforming growth factor-β (TGFβ), and other cytokines to promote tumor growth and progression 10. Moreover, mMDSCs can further differentiate into tumor-associated macrophages (TAMs) and promote the immunosuppressive function of regulatory T cells (Tregs) 11. Therefore, identification of molecular pathways that influence the immunosuppressive activity of MDSCs or inhibit accumulation of MDSCs in the TME will provide new approaches for improving the response to immunotherapy.

Receptors with the Fc region of immunoglobulins (Igs) play an essential role in the activation and/or inhibition of immune responses 12. Fc gamma receptor IIB (FcγRIIB/CD32B) is the only inhibitory member of the Fc gamma receptor family expressed on the B cells, macrophages, dendritic cells (DCs), and granulocytes 13. The intracellular domain of this receptor contains an immunoreceptor tyrosine-based inhibitory motif (ITIM) that recruits the inhibitory phosphatase SHIP, which inhibits the phosphorylation of downstream signaling molecules involved in the activation of monocytes, macrophages, and DCs 14. Activation of this receptor reduces the cell proliferation and antibody production, and may also deliver apoptotic signals 15. Moreover, FcγRIIB is a negative regulator of antibody production and inflammatory responses 16. Genetic deficiency of FcγRIIB was shown to enhance tumor-infiltrating CD8^+^ T cell responses and reduce tumor burden 17. However, the expression and function of FcγRIIB in MDSCs have not yet been studied.

Here, we aimed to investigate the role of FcγRIIB on MDSCs during cancer development, and explore new anti-cancer approaches by targeting FcγRIIB.

## Results

### Expression of FcγRIIB is elevated in tumor-infiltrating MDSCs

**Figure 1:**
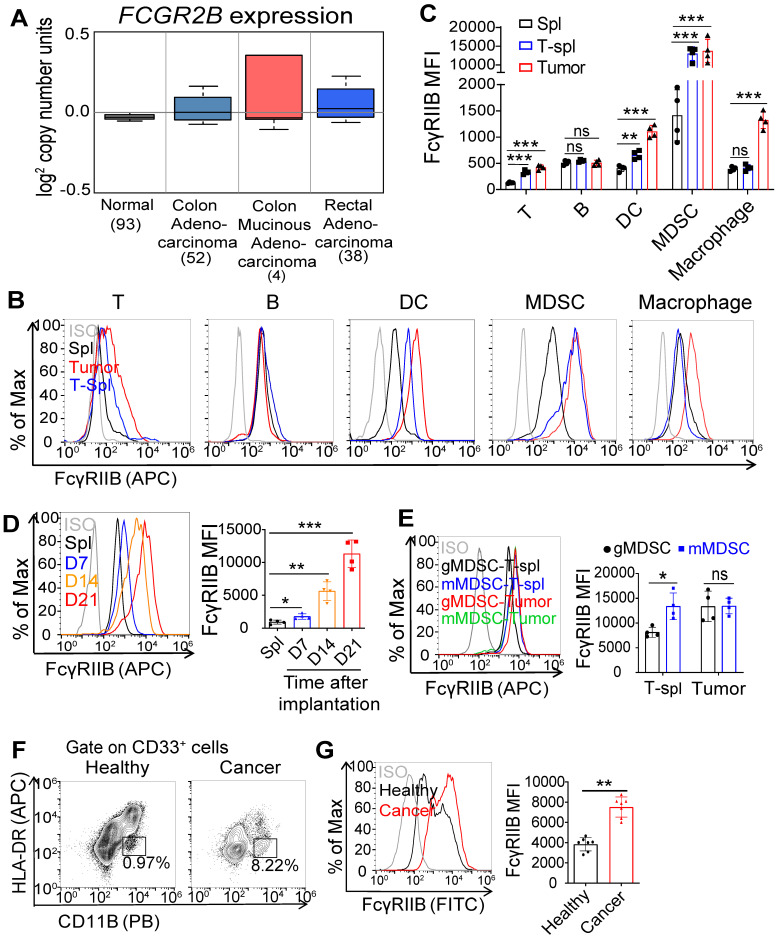
** Increased FcγRIIB expression on tumor-infiltrating MDSCs. (A)** Gene expression of *FCGR2B* in normal colon, colon adenocarcinoma, colon mucinous adenocarcinoma and rectal adenocarcinoma tissues according to Kurashina Colon cancer in Oncomine datasets. **(B)** Representative histogram plots of the expression of FcγRIIB in T cells, B cells, DCs, MDSCs and macrophages from splenocytes of tumor free mice (Spl), MC38 tumor tissues (Tumor) and splenocytes of tumor bearing mice (T-Spl), ISO, isotype control. **(C)** The expression of FcγRIIB for T cells, B cells, DCs, MDSCs and macrophages, as shown in (**B**), MFI is shown, *n* = 4. **(D)** The expression of FcγRIIB for MDSCs (CD11b^+^Gr1^+^) in the splenocytes of 100 μL PBS injected C57BL/6 mice for 21days (Spl) or MC38 tumor tissues were quantified at different time points (7, 14 and 21days) after MC38 tumor cell inoculation (*n* = 4). **(E)** FcγRIIB expression in gMDSCs (CD11b^+^Ly6G^+^Ly6C^low^) and mMDSCs (CD11b^+^Ly6C^high^Ly6G^-^) from the MC38 tumor bearing spleens (T-spl) or MC38 tumor tissues were measured by FCM, *n* = 4. **(F, G)** Representative flow cytometry plots (**E**) and FcγRIIB expression **(F)** of MDSCs in the peripheral blood of patients with CRC *versus* healthy donors (*n* = 7). Data are expressed as means ± SD. ^*^P < 0.05, ^**^P < 0.01, ^***^P < 0.001, Mann-Whitney test were used for all comparisons, *ns,* no significant difference.

We analyzed the expression of FcγRIIB in human colorectal cancer (CRC) tissues using Kurashina Colon cancer datasets in Oncomine and found that FcγRIIB expression was modestly increased in the CRC tissues than that in normal tissues (Figure 1A). Next, we examined FcγRIIB expression in tumor tissues of MC38 tumor-bearing mice. Consistent with results of previous reports 18, 19, FcγRIIB was expressed in several cell types, including the B cells, dendritic cells (DCs), and macrophages (Figures 1B, 1C and S1). Of note, 10-fold increase of FcγRIIB was observed in tumor-infiltrating MDSCs than that in other myeloid-derived cells. In addition, the expression of FcγRIIB on MDSCs increased with tumor progression (Figure 1D). Further analysis revealed that both mMDSCs and gMDSCs expressed high levels of FcγRIIB in the TME, among which, mMDSCs showed higher levels of FcγRIIB than gMDSCs in the spleen of MC38 tumor-bearing mice (Figure 1E). An increased FcγRIIB expression was also observed in MDSCs from the peripheral blood from CRC patients (Figures 1F and 1G). These results demonstrate that FcγRIIB is elevated in tumor-infiltrating MDSCs during tumorigenesis.

### Deficiency of FcγRIIB impairs MDSC accumulation and tumorigenesis

**Figure 2:**
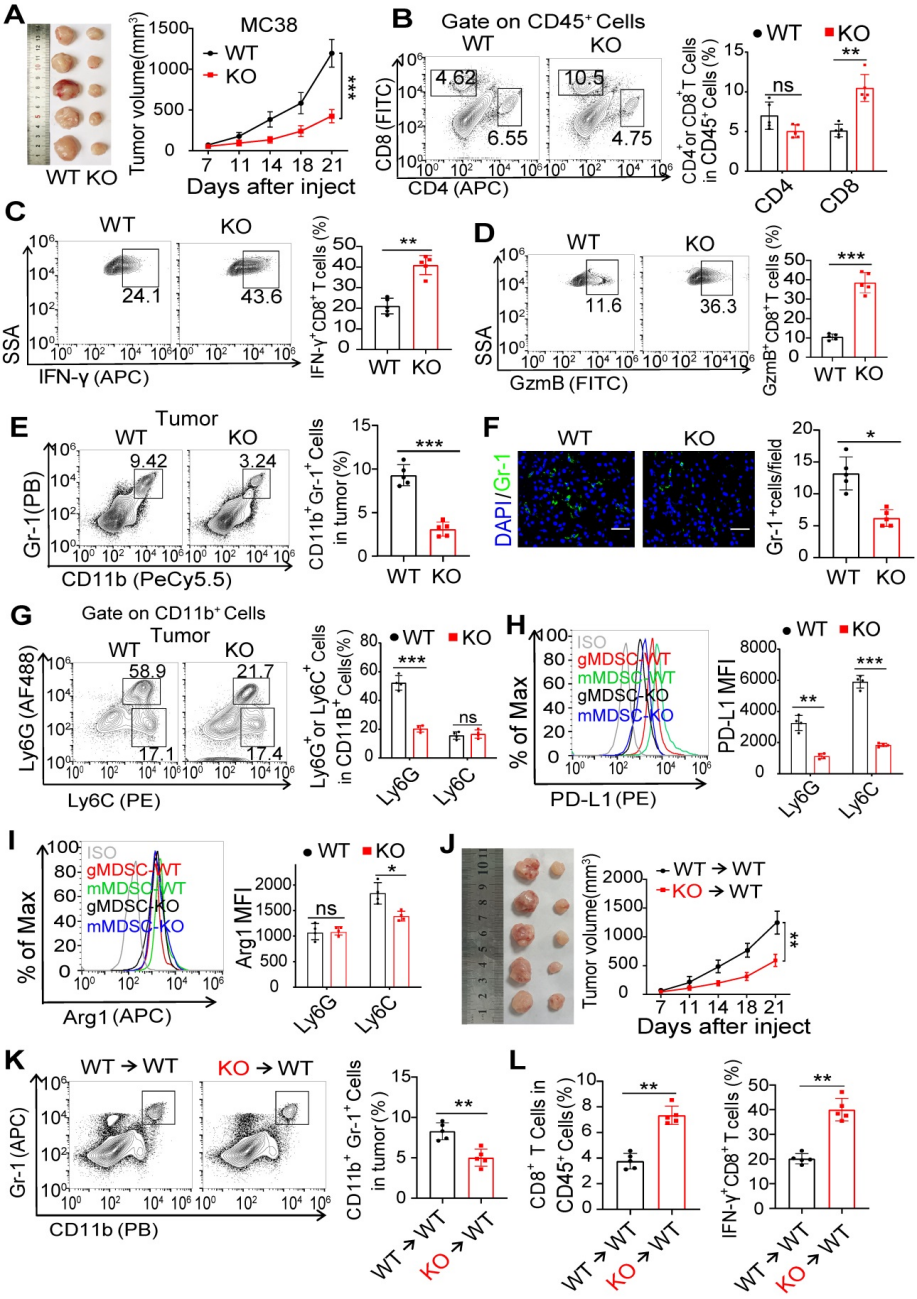
** FcγRIIB deficiency in MDSCs inhibits tumorigenesis. (A)** C57BL/6 (WT) or FcγRIIB**^-/-^** (KO) mice were injected subcutaneously with 10^6^ MC38 cells. Tumor growth was monitored at indicated time points, *n* = 5. **(B)** WT or KO mice were sacrificed at day 14 post-xenograft of MC38 cells and tumors were analyzed by FCM for the frequency of CD4^+^ and CD8^+^ T cells. Shown data are representative from two independent experiments, *n* = 5 per group. **(C, D)** The frequency of IFN-γ-producing **(C)** and GzmB-producing **(D)** CD8^+^ T cells in the MC38 tumor from WT and FcγRIIB-KO mice were determined by FCM. **(E)** The frequency of tumor-infiltrating MDSCs in WT or KO tumor was assessed 14 days after MC38 tumor inoculation. **(F)** The expression of Gr-1 (green) in MC38 tumor tissue sections from WT or KO mice was detected by immunofluorescence. Scale bars: 50 μm. **(G)** gMDSCs and mMDSCs frequency in CD11b^+^ cells from WT or KO tumor tissues were analyzed by FCM. **(H, I)** PD-L1** (H)** and Arg1 **(I)** expression in gMDSCs and mMDSCs from WT or KO tumor tissues were analyzed by FCM; *n =* 4. **(J-L)**, Irradiated WT mice (CD45.1) were *i.v.* injected with WT (CD45.2) or FcγRIIb**^-/-^** (CD45.2) BM Cells for BM reconstitution assay. Six weeks after BM chimaera reconstitution, mice were injected subcutaneously with 1 × 10^6^ MC38 cells. Tumor size was monitored over time **(J)**, *n =* 5; The frequency of tumor-infiltrating MDSCs, *n =* 5 **(K)**, CD8^+^ T cells and IFN-γ-producing CD8^+^ T cells, *n =* 5 **(L)** in tumor from BM chimeric mice was assessed. Data are expressed as means ± SD. ^*^P < 0.05, ^**^P < 0.01, ^***^P < 0.001, by Mann-Whitney test.

To assess the role of FcγRIIB on tumorigenesis, the MC38 cells were subcutaneously inoculated into wild-type (WT) and FcγRIIB^-/-^ (KO) mice. The KO mice showed slower growth of xenograft tumors and prolonged survival than WT mice (Figures 2A and S2A). The tumor growth was also dampened in KO mice implanted with B16F10 melanoma cells than in WT mice (Figure S2B). Tumor-bearing mice always show splenomegaly at later stages of tumorigenesis 20. We found that the spleen size in tumor-bearing KO mice was smaller than that in tumor-bearing WT mice, while it was comparable to that in tumor-free WT mice (Figure S2C).

CD8^+^ T cells are pivotal for controlling tumor growth 21. We found that the percentages of tumor-infiltrating CD8^+^ T cells were increased in KO mice than that in WT mice (Figures 2B and S2D-F). Moreover, the proportions of interferon γ (IFNγ) and granzyme B (GzmB)-producing CD8^+^ T cells were significantly increased in KO mice than that in WT mice, suggesting that compared with WT mice, the anti-tumor immunity was enhanced in the FcγRIIB deficient mice (Figures 2C and 2D).

In the TME, immunosuppressive cells, including MDSCs, tumor-associated macrophages (TAMs), and Tregs suppress the activity of CD8^+^ T-cells 22. We determined the infiltration of these immunosuppressive cells into the TME, and found that MDSC accumulation was reduced in the tumor and spleen of MC38 tumor-bearing FcγRIIB-KO mice than in that of tumor-bearing WT mice (Figures 2E, 2F, and S2D-H), whereas there was no significant difference in the levels of Treg cells, B cells, and DCs (Figures S2D-E). Similarly, decreased MDSC accumulation were also observed in B16F10 tumor-bearing FcγRIIB-KO mice (Figure S2I). Interestingly, the percentages of TAMs were higher in KO tumors than in WT control. Compared with WT TAMs, the FcγRIIB-KO TAMs exhibited increased M1 markers CD86 and MHC II, whereas the expression of CD80 showed no significant difference (Figure S2J). Additionally, the proportion of mMDSC subpopulations remained unchanged, but that of gMDSCs decreased in FcγRIIB deficient mice (Figures 2G and S2D), indicating that the major decreased proportion of MDSCs in KO mice was gMDSCs. The mMDSCs and gMDSCs from FcγRIIB-KO mice expressed decreased PD-L1, and mMDSCs expressed slightly decreased arginase-1 (Arg-1) (Figures 2H, 2I and S2K).

To investigate whether the reduced tumor burden in KO mice was due to the loss of FcγRIIB in MDSCs, we reconstituted irradiated mice with bone marrow (BM) cells isolated from WT or FcγRIIB KO littermates (Figure S2L). We found that KO→WT chimeric mice exhibited reduced tumor growth when compared with WT→WT chimeric mice (Figure 2J). Interestingly, significantly fewer MDSCs, while abundant IFNγ^+^CD8^+^ T cells, were found in the tumor tissues from KO→WT BM chimeric mice than WT→WT BM chimeric mice (Figures 2K and 2L). These data demonstrate that FcγRIIB deficiency in MDSCs inhibits tumor development by reducing accumulation of gMDSC in the TME.

### FcγRIIB-dependent immunosuppressive activity of MDSCs

**Figure 3:**
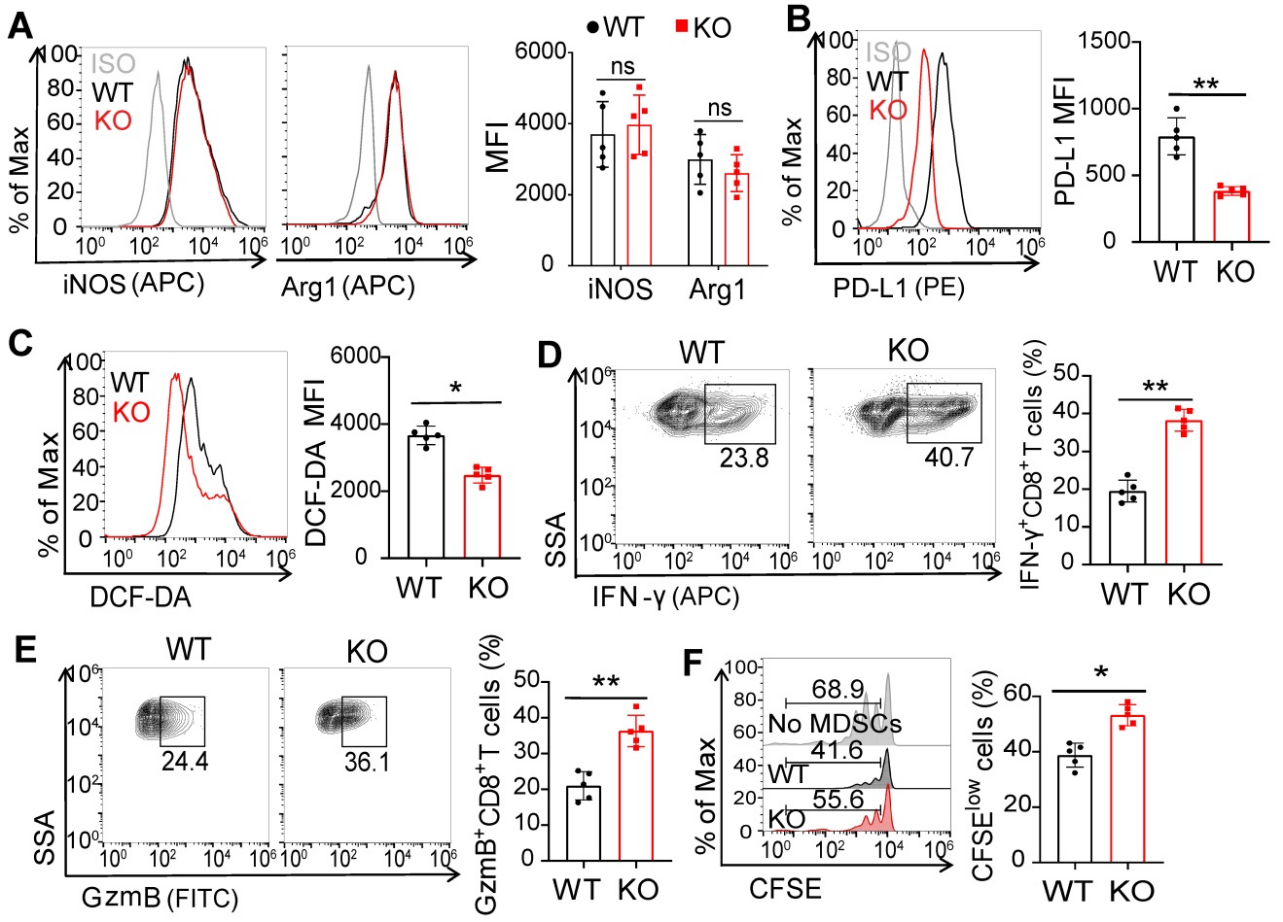
** FcγRIIB deficiency suppresses the immunosuppressive activity and differentiation of MDSCs. (A)** iNOS and Arg1 expression in WT and KO MDSCs from MC38 tumors were assessed using FCM; *n =* 5. **(B, C)** PD-L1 **(B)** and DCF-DA **(C)** expression in WT and KO MDSCs; *n =* 5. **(D**-**F)** Isolated MDSCs from MC38 tumors of WT and KO mice were cocultured with CFSE-labeled CD8^+^ T lymphocytes isolated from WT mice (1:2) for 3 days, the production of IFN-γ **(D)**, and GzmB **(E)** in CD8^+^ T cells, anti-CD3 and anti-CD28 induced proliferation** (F)** were measured by FCM; *n =* 4. Data are expressed as means ± SD. ^*^P < 0.05, ^**^P < 0.01, *ns,* no significant difference, by Mann-Whitney test.

We next wondered whether FcγRIIB regulated the immunosuppressive activity of MDSCs. The levels of iNOS and Arg-1 showed no significant change in MDSCs from WT and FcγRIIB-KO tumor-bearing mice (Figure 3A). However, the expression level of PD-L1 was decreased in MDSCs from FcγRIIB-KO mice than those from WT mice (Figure 3B). Excessive ROS production is another well-known mechanism for MDSC-mediated immunosuppression 8. We found that intracellular ROS level was significantly decreased in KO MDSCs than that in WT MDSCs (Figure 3C). Additionally, the activation of CD8^+^ T cells was significantly augmented in FcγRIIB-KO than in WT mice (Figures 3D and 3E). Moreover, KO MDSCs also increased the proliferation of CD8^+^ T cells than WT MDSCs (Figure 3F). These results indicate that FcγRIIB promotes the immunosuppressive activity of MDSCs that contributes to tumor immunoescape.

### FcγRIIB is required for gMDSC differentiation from HPCs

**Figure 4:**
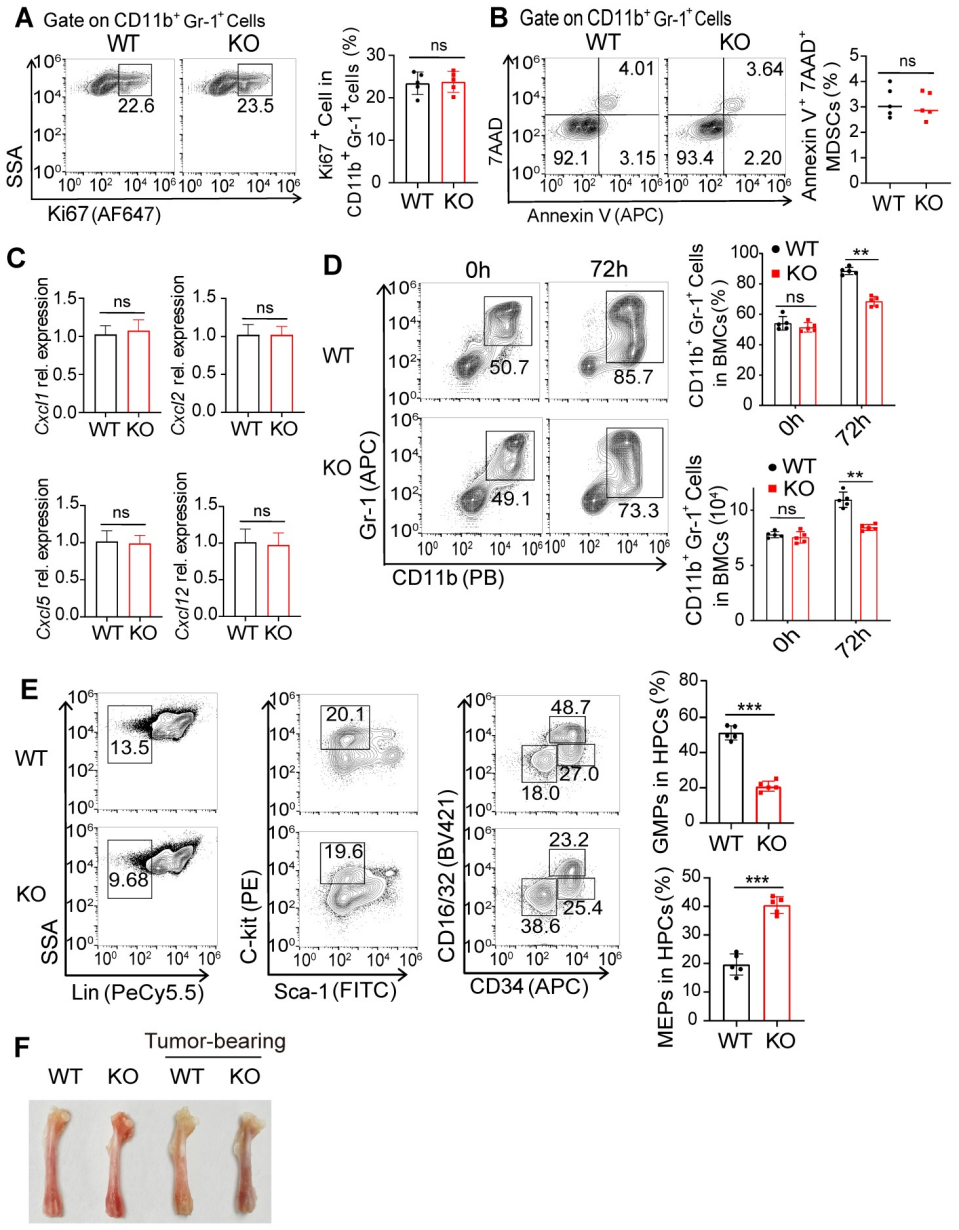
** FcγRIIB deficiency impairs the differentiation of gMDSCs from HPCs in the tumor-bearing mice. (A)** The percentage of proliferating (Ki67^+^) MDSCs in WT and KO tumor tissues were analyzed with flow cytometry after Ki67 staining, *n =* 5. **(B)** Representative staining and frequencies of AnnexinV^+^7AAD^+^ cells in MDSCs from WT and KO tumor tissues were assessed by FCM. **(C)** The gene expression of MDSC-related chemokines within whole tumor tissues from WT and KO mice were detected by qPCR;* n =* 4. Data are expressed as means ± SD. **(D)** WT and KO BMs were treated with GM-CSF/IL-6 (20ng/mL) to induce MDSCs differentiation, MDSCs ratio and numbers were analyzed after 72 hrs. **(E)** Gating strategy for granulocyte/macrophage progenitors (GMP; Lin^-^Sca-1^-^C-kit^+^CD16/32^+^CD34^+^), common myeloid progenitors (CMP; Lin^-^Sca-1^-^C-kit^+^CD16/32^int^CD34^+^), megakaryocyte/erythrocyte progenitors (MEP; Lin^-^Sca-1^-^C-kit^-^CD16/32^-^CD34^-^), and percentages of these HPCs subpopulations rates in BMs from WT and KO tumor bearing mice were detected, *n =* 5. **(F)** Representative photograph of femurs dissected from WT and KO tumor-free or MC38 tumor-bearing mice on day 21 after tumor cells implantation. Data are expressed as means ± SD. ^*^P < 0.05, ^**^P < 0.01, ^***^P < 0.001, *ns,* no significant difference, by Mann-Whitney test.

As FcγRIIB-deficient mice showed decreased numbers of tumor-infiltrating gMDSCs, the roles of FcγRIIB on the proliferation, apoptosis, or chemotaxis in MDSCs were sought. However, no significant difference in the proliferation and apoptosis of MDSCs was observed between WT and KO mice (Figures 4A and 4B). Next, we analyzed MDSC chemotaxis-related genes, including *Cxcl1*, *Cxcl2*, *Cxcl5*, and *Cxcl12* in tumor tissues. The data indicated no difference in levels of these genes between WT and KO mice, suggesting that decreased numbers of MDSCs in the tumors may not be due to decreased chemotaxis (Figure 4C).

MDSCs are differentiated from HPCs and subsequent granulocyte/macrophage progenitors (GMP) by induction of GM-CSF 1, 23. We treated BM cells with GM-CSF and IL-6 for 72 h, and found that GM-CSF treatment induced the expansion of MDSCs (Figure 4D). Moreover, FcγRIIB-KO BM cells yielded reduced numbers of MDSCs than WT, suggesting that FcγRIIB may contribute to the generation of MDSCs. Consistently, KO BM cells had decreased proportions of GMPs, but increased megakaryocyte/erythrocyte progenitors (MEP) within the HPC population than WT BM (Figure 4E). Furthermore, the bones from tumor-bearing WT mice were paler than those from tumor-bearing KO mice (Figure 4F), suggesting that erythropoiesis was dampened in WT tumor-bearing mice. The GMP subpopulation was also decreased in HPCs from KO naive mice than that from WT mice, indicating an intrinsic role of FcγRIIB in the differentiation of BM progenitors into GMP. Additionally, the GMP subpopulation was increased in tumor-bearing WT mice, while the expansion of GMPs was decreased in KO HPCs (Figures S3A and S3B).

As a member of Fc receptor family, immunoglobulins, pentraxins such as Serum Amyloid protein (SAP) and C Reactive Protein (CRP) can bind to FcγRIIB 24, 25. To determine whether the differentiation of HPCs to MDSCs induced by FcγRIIB was functionally due to immunoglobulins, SAP or CRP, WT and FcγRIIB KO BM cells were stimulated with GM-CSF and IL-6 for 72h in the presence or absence of IgG, SAP or CRP. We found that IgG, SAP or CRP administration had no impact on expansion of MDSC populations (Figures S3C and S3D), indicating that IgG, SAP and CRP are not required for FcγRIIB-mediated differentiation of HPCs. The immunosuppressive cytokine, soluble fibrinogen-like protein 2 (sFgl2), is also a ligand of FcγRIIB, and possesses immune regulatory functions *via* the FcγRIIB pathway 26. However, mice deficiency of *Fgl2* had no effect on the composition of GMPs in HPCs (Figure S3E), suggesting that FcγRIIB-mediated HPC differentiation independent of Fgl2. Taken together, these results indicate that FcγRIIB is required for the differentiation of HPCs into GMPs, which increases the levels of tumor-infiltrating gMDSCs.

### Stat3 is involved in FcγRIIB-mediated differentiation of HPCs into gMDSCs

**Figure 5:**
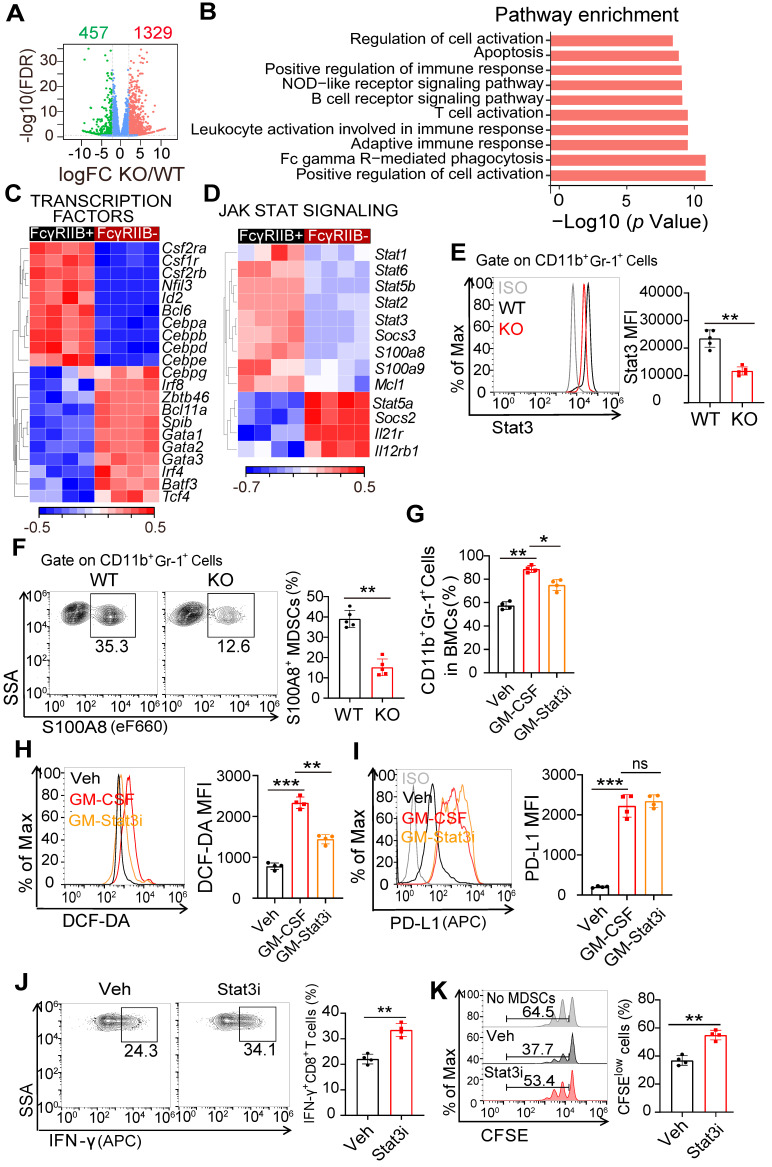
** FcγRIIB promotes gMDSCs generation from HPCs *via* Stat3 signaling. (A)** Volcano plots of differentially expressed genes in HPCs from WT or FcγRIIB KO mice at 14 days post-grafting of MC38 cells (adjusted *P* ≤ 0.01 and fold change (FC) ≥ 2), *n =* 4. **(B)** Signaling pathway enrichment analysis of differentially expressed genes. **(C)** Heatmap of differentially expressed genes that function as transcription factors involved in differentiation of HPCs into GMPs, *n =* 4. **(D)** Heatmap of differentially expressed genes in Jak-Stat signaling from WT or FcγRIIB KO HPCs, *n =* 4. **(E, F)** WT or KO Mice were sacrificed at day 14 post-grafting, and tumor-infiltrating MDSCs were assessed for Stat3 **(E)** and S100A8** (F)** expression; *n =*5. **(G)** WT mice BMs were treated with Veh (PBS), GM-CSF (20ng/mL) for 48 hrs, in the presence or absence of STAT3-IN-1 (GM-Stat3i, 1μM), and MDSCs proportion were analyzed. **(H, I)** Isolated Gr-1^+^ cells from BM were treated with GM-CSF (20ng/mL) for 48 hrs, in the presence or absence of STAT3-IN-1 (GM-Stat3i, 1μM), DCF-DA **(H)** and PD-L1 (**I**) expression were assessed using FCM; *n =* 4. **(J, K)** BM derived MDSCs were treated with STAT3-IN-1 (GM-Stat3i, 1μM) for 48 hrs, and then cocultured with CFSE-labeled CD8^+^ T lymphocytes isolated from WT mice (1:2) for 3 days, the production of IFN-γ in CD8^+^ T cells **(J**, *n =* 4**)**, anti-CD3 and anti-CD28 induced proliferation **(K**, *n =* 4**)** were measured by FCM; *n =* 4. Data are expressed as means ± SD. ^**^P <0.01, by Mann-Whitney test.

To elucidate the molecular mechanisms underlying FcγRIIB deficiency-mediated differentiation of HPCs, we performed RNA sequencing (RNA-seq) in HPCs from WT and FcγRIIB-KO tumor-bearing mice. A total of 1,786 genes were differentially expressed between WT and KO HPCs (Figure 5A). Subsequent analysis of these genes identified enrichment in cell activation and immune response pathways (Figure 5B). A number of transcription factors that control differentiation of common myeloid progenitors (CMPs) to GMPs were also assessed between the WT and KO groups (Figure 5C). JAK/STAT pathways have critical roles in MDSC differentiation and function 27. The analysis showed that the expression of Stat1, Sat2, Stat3 and Stat5b were significantly lower in FcγRIIB-deficient HPCs than that in WT HPCs (Figures 5D and 5E). Among these Stats, Stat3 is one of the most well-known protein that associated with MDSC differentiation and immunosuppressive functions. Stat3-mediated upregulation of S100A8/9 promotes accumulation of MDSCs in cancer 28. Consistently, the expressions of S100A8 and S100A9 were lower in KO HPCs and MDSCs than in WT (Figures 5D-F), indicating that FcγRIIB activates Stat3 signaling. Furthermore, blocking Stat3 signaling inhibited GM-CSF-induced expansion of MDSCs, reduced expression of ROS in MDSCs, and enhanced the activation and proliferation of CD8^+^ T cells (Figures 5G-K). In contrast, IL-6-upregulated PD-L1 and ROS were remarkably abrogated by the absence of FcγRIIB (Figures S4A and S4B). Moreover, gene set enrichment analysis (GSEA) also revealed that FcγRIIB-KO HPCs, but not WT HPCs, were positively enriched in HALLMARK gene sets for erythrocyte development, erythrocyte differentiation, and erythrocyte homeostasis signaling pathways, indicating that deficiency of FcγRIIB facilitates differentiation of HPCs to megakaryocyte/erythrocyte-restricted progenitors (MEPs) but not GMPs (Figure S4C). Although other Stats may also participate in the MDSC differentiation, these results suggest that deficiency of FcγRIIB reduces the differentiation of HPCs into gMDSCs, at least partially through the Stat3 signaling.

### Tumor cell-derived GM-CSF induces expression of FcγRIIB during MDSC differentiation *via* the Sp1 signaling

**Figure 6:**
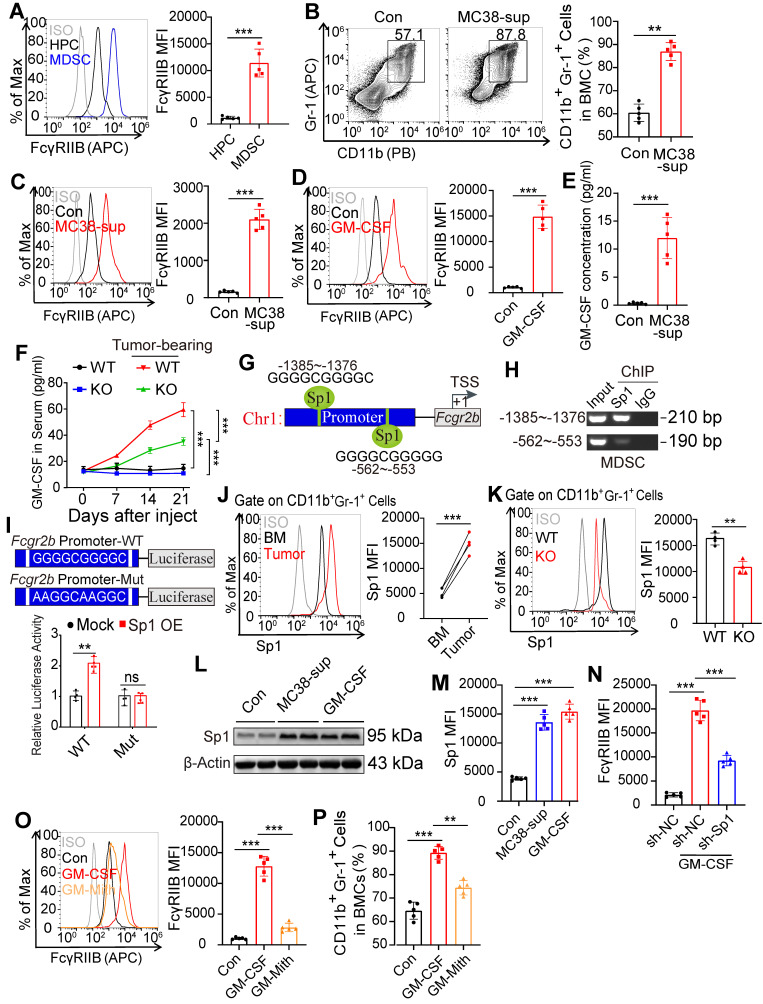
** GM-CSF induces FcγRIIB expression on MDSCs *via* Sp1 signaling. (A)** FcγRIIB expression on HPCs and tumor-infiltrating MDSCs were analyzed, representative histogram plots were shown. **(B, C)** WT and KO BM cells were treated with 10% tumor supernatants (from MC38 cells) for 48 hrs, the percentage of CD11b^+^Gr-1^+^ cells in BM cells **(B)**, FcγRIIB expression on MDSCs **(C)** was determined. **(D)** WT mice BM cells were treated with PBS (Control) or GM-CSF (20 nM) for 48 hrs, the expression of FcγRIIB were determined with flow cytometry. **(E)** GM-CSF protein levels in supernatants of MC38 tumor were measured using ELISA, *n =*5. **(F)** GM-CSF protein levels in serum of tumor-free or MC38 tumor-bearing WT and FcγRIIb^-/-^ mice were measured using ELISA, *n =* 4 each group. **(G)** *In silico* analysis predicted two Sp1 binding site in the promoter of *Fcgr2b*, TSS; transcription start site. **(H)** ChIP assay analyzed recruitment of Sp1 to *Fcgr2b* gene locus in WT MDSCs. The prepared chromatin from MDSCs was immunoprecipitated with an anti-Sp1 antibody or control IgG, and pulled-down DNA was subjected to qPCR using the specific primers designed for Sp1 binding region. **(I)** Luciferase report assay of *Fcgr2b* promoter containing WT or mutant Sp1 binding site in Sp1 overexpression HPCs. **(J)** Sp1 expressions in CD11b^+^Gr-1^+^ cells from BM and paired tumor were measured by FCM,* n =* 5. **(K)** Sp1 expression in WT and KO MDSCs were measured by FCM,* n =* 4. **(L**, **M)** CD11b^+^Gr-1^+^ cells from BM were treated with 10% tumor supernatants or GM-CSF (20 nM) for 48 h, the expression of Sp1 was determined by western blot **(L)** and FCM **(M)**, *n =* 5. **(N)** CD11b^+^Gr-1^+^ cells from BM were transfected with control (sh-NC) or virus expressing shRNA against Sp1 (sh-Sp1), in the presence or absence of 20 nM GM-CSF for 48 hrs, FcγRIIB expression on MDSCs were analyzed. **(O, P)** BM cells were treated with GM-CSF for 48 hrs, in the presence or absence of 1 μL PBS (Con) or Mithramycin A (Mith, 20 nM), FcγRIIB expression on MDSCs **(O)** and the percentages of MDSCs **(P)** in BM cells were analyzed by FCM. Data are expressed as means ± SD. ^**^P <0.01, by Mann-Whitney test (**A** to** F, I, K** to** P**) or Wilcoxon matched-pairs signed rank test (**J**).

We next investigated the involvement of FcγRIIB in the differentiation of HPCs. We found that the expression of FcγRIIB was significantly elevated in MDSCs than that in HPCs (Figure 6A), suggesting that FcγRIIB was upregulated during differentiation. Further, stimulation of BM cells with supernatants from MC38 cancer cells led to significantly increased proportion of MDSCs (Figure 6B), along with increased expression of FcγRIIB in MDSCs (Figure 6C).

GM-CSF is an essential cytokine for expansion of MDSCs and can be secreted by tumors or tumor-infiltrating immune cells 29. Treatment with GM-CSF induced expression of FcγRIIB on HPCs (Figure 6D). The supernatants from MC38 cancer cells also exhibited increased level of GM-CSF (Figure 6E) suggesting that GM-CSF may be responsible for upregulation of FcγRIIB and generation of MDSCs. Consistently, tumors bearing mice exhibited increased serum and BM concentrations of GM-CSF with tumor progression (Figures 6F, S5A, and S5B), suggesting that tumor cell-derived GM-CSF induces FcγRIIB expression to contribute to MDSC differentiation.

To elucidate how GM-CSF regulated FcγRIIB, we analyzed the potential transcription factors that bind to the promoter of *Fcgr2b* using the online tool ConTra V3 30. The *in silico* analysis predicted two binding sites for Sp1 within the *Fcgr2b* promoter (Figure 6G). Sp1 is a zinc-finger transcription factor that binds to GC-rich motifs to regulate the expression of genes involved in proliferation, apoptosis, differentiation, and immune responses 31. The ChIP-qPCR results showed that the first DNA fragment containing the Sp1 response element could be amplified from the Sp1-immunoprecipitated samples, suggesting that this GC-rich motif may be critical for interaction with Sp1 (Figure 6H). Luciferase report assay data showed that the reporter activity of *Fcgr2b* was activated by Sp1 over-expression, whereas mutation of predicted Sp1 binding site attenuated the ability of Sp1 to activate *Fcgr2b* promoter activity (Figure 6I). Moreover, expression of Sp1 was upregulated in tumor-infiltrating MDSCs and was decreased in FcγRIIB KO MDSCs (Figures 6J and 6K). The tumor supernatants and treatment with GM-CSF also significantly upregulated the expression of Sp1 (Figures 6L and 6M). Knockdown (KD) of Sp1 or mithramycin A (Mith; Sp1 inhibitor) treatment decreased the expression of FcγRIIB in mouse MDSCs (Figures 6N and 6O) and human THP1 monocytes (Figure S5C) 31. Mith treatment inhibited GM-CSF-induced expansion of MDSCs (Figures 6P and S5D). Csf2R is a receptor of GM-CSF that controls the differentiation of the myeloid lineage 32. Interestingly, we found that the expression of GM-CSF receptors (Csf2Ra and Csf2Rb) in FcγRIIB-KO HPCs were lower than that in WT HPCs (Figure 5C), suggesting a potential positive loop between GM-CSF and FcγRIIB that promoted the accumulation of immunosuppressive MDSCs. These data suggest that tumor cell-derived GM-CSF activates Sp1 signaling, leading to the upregulation of FcγRIIB and differentiation of MDSCs from HPCs.

### Blocking Sp1-FcγRIIB signaling dampens immunosuppressive activity of MDSCs and tumor progression

We next assessed the role of FcγRIIB on tumor growth. The FcγRIIB specific antagonistic monoclonal antibody treatment suppressed MC38 tumor growth and the percentage of tumor-infiltrating MDSCs. Similar results were observed by anti-Gr-1 neutralizing antibody treatment (Figures S6A and S6B). However, combined treatment with anti-Gr-1 neutralizing antibody and FcγRIIB antagonistic antibody did not induce further reduction of tumor volume and tumor-infiltrating MDSCs compared with mice treated with FcγRIIB antagonistic antibody alone (Figures S6A and S6B). FcγRIIB blockade also led to increased proportions of CD8^+^ T cells in the tumor and enhanced CD8^+^ T cell activation (Figures S6C and S6D). These data demonstrate that FcγRIIB is required for the immunosuppressive activity of MDSCs *in vivo*.

**Figure 7:**
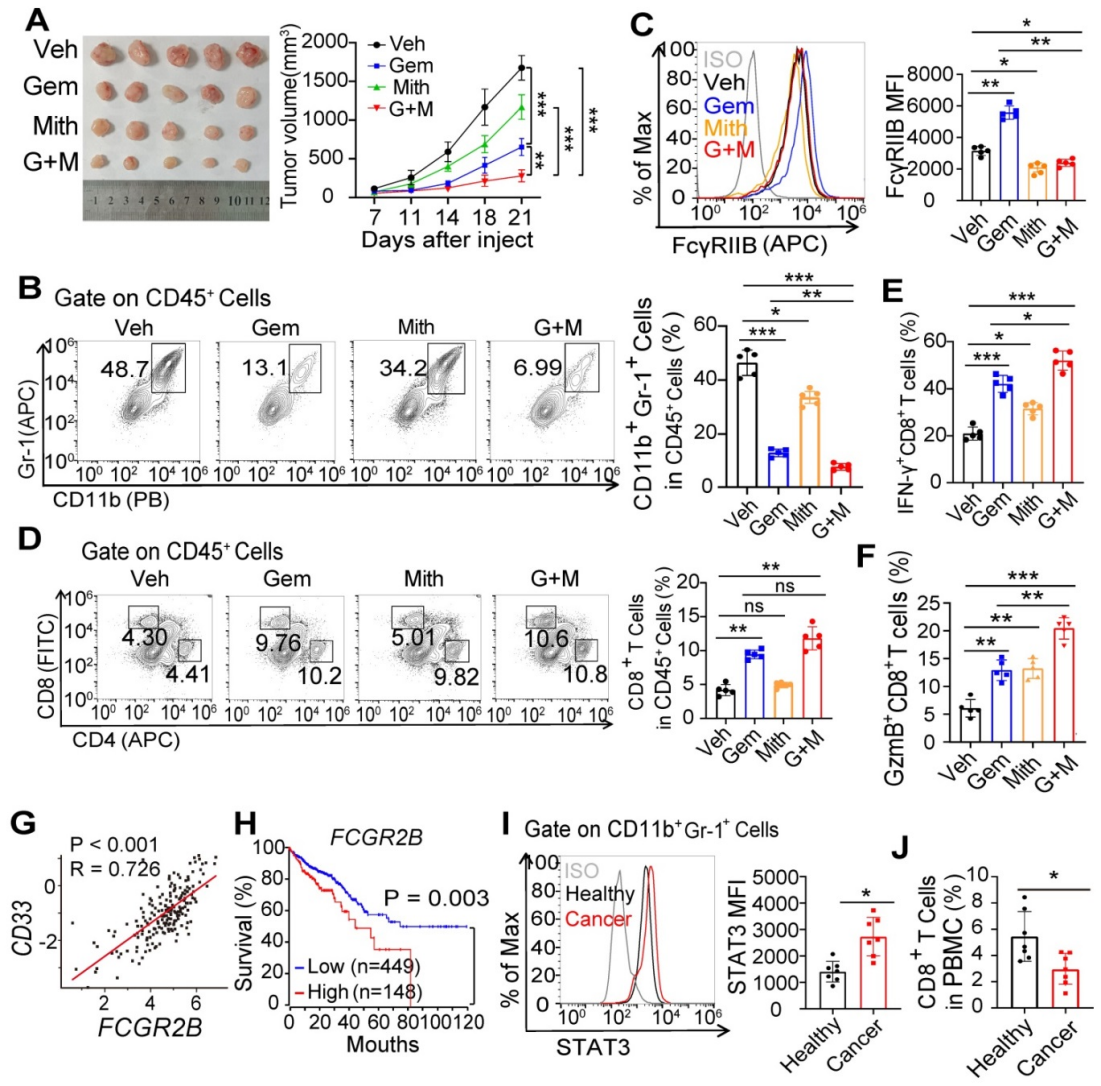
** Inhibition of FcγRIIB expression improves the anti-tumor effect of gemcitabine. (A**-**C)** WT mice were injected subcutaneously with MC38 tumor cell. After 7 days, tumor-bearing mice were injected with PBS (Veh), gemcitabine (Gem, 50 mg/kg), Mithramycin A (Mith, 0.2mg/kg) or combined Gem with Mith (G+M). Tumor growth was monitored for 21 days **(A)**. All mice were euthanized on day 21 after tumor injection, the percentages of MDSCs **(B)** and FcγRIIB expression on MDSCs **(C)** in tumor tissues were analyzed by FCM. **(D**-**F)** The percentages of CD8^+^ T cells in tumor tissues **(D)**, the percentage of CD8^+^ T cells producing IFN-γ **(E)** and GzmB **(F)** in tumor tissues were analyzed by FCM. **(G)** Pearson's correlation coefficient was used to determine the correlation between FCGR2B and CD33. **(H)** Colon adenocarcinoma patient survival data were obtained from TCGA database, and overall survival probability was calculated using the Kaplan-Meier analysis, and the differences in survival curves were assessed using the log-rank test. **(I, J)** STAT3 expression in MDSCs (**I**) and CD8^+^ T cells percentages **(J)** in peripheral blood of patients with CRC versus healthy donor were analyzed by FCM, *n* = 7. Data are shown as means ± SD. One way ANOVA with Tukey multiple comparison post-test was used to evaluate statistical significance. ^*^P < 0.05, ^**^P < 0.01, ^***^P < 0.001. *ns,* no significant difference.

Clinically, chemotherapeutic drugs including gemcitabine and 5-FU can selectively deplete levels of MDSCs and restore immune surveillance 33. We found that treatment with gemcitabine inhibited the accumulation of MDSCs and delayed tumor progression (Figures 7A and S7A). Interestingly, expression of FcγRIIB in mMDSCs was increased after treatment with gemcitabine (Figure S7B). We therefore speculated whether FcγRIIB inhibition by Sp1 inhibitor might have a synergistic effect with gemcitabine to inhibit tumor progression. Indeed, tumor growth was significantly suppressed by combined treatment of gemcitabine with Mith (Figure 7A). The proportion of tumor-infiltrating MDSCs was further decreased in mice in the combined treatment group than that in single treatment group (Figure 7B). As expected, treatment with Mith inhibited expression of FcγRIIB on MDSCs (Figure 7C). Moreover, inhibition of FcγRIIB led to enhanced infiltration and activation of CD8^+^ T cells in gemcitabine-treated tumor-bearing mice (Figures 7D-F), suggesting that suppression of FcγRIIB could boost the anti-tumor response of chemotherapy.

Finally, TCGA analysis showed that the expression of *FCGR2B* positively correlated with *CD33*, a marker of MDSCs (Figure 7G), and that higher *FCGR2B* expression was associated with poor survival in patients with CRC (Figure 7H). We also observed increased STAT3 expression in MDSCs and decreased CD8^+^ T cells percentages in peripheral blood of patients with CRC **(**Figures 7I and 7J**)**. Taken together, these results indicate that blockade of Sp1 is an efficient anti-tumor approach *via* reducing FcγRIIB-mediated accumulation and immunosuppressive activity of MDSCs in the TME.

## Discussion

MDSCs are a heterogeneous group of immature HPCs that function as pivotal immunosuppressors in the TME. Multiple evidence indicates that depletion of MDSCs by treatment with anti-Gr-1 neutralizing antibody or gemcitabine can restore immune surveillance and improve the efficacy of cancer immunotherapies *in vivo* 10, 34. In the present study, we found that FcγRIIB was highly expressed on tumor-infiltrating MDSCs. Deficiency or blockade of FcγRIIB decreased the accumulation and immunosuppressive activity of MDSCs in the TME. Tumor cell-derived GM-CSF increased the expression of FcγRIIB on MDSCs, along with enhanced differentiation of MDSCs from HPCs by activating the Sp1 signaling. Inhibition of Sp1 signaling significantly reversed GM-CSF-induced expression of FcγRIIB and the immunosuppressive activity of MDSCs, while synergistically enhanced gemcitabine-suppressed tumorigenesis. These findings indicate the critical role of GM-CSF/Sp1/FcγRIIB signaling pathway in tumor immunity and suggest potential therapeutic targets.

FcγRIIB is the most widely expressed inhibitory Fcγ receptor in both human and mice. In previous studies, FcγRIIB expression in DCs has been reported can inhibit T cell response while anti-FcγRIIB antibody treatment results in up-regulation of IFN-induced genes 35, 36. FcγRIIB has also been shown to negatively regulate cytotoxicity of NK cells, and antibody-producing B cells 37. Recent study reported that FcγRIIB plays a cell-intrinsic role in suppressing tumor-infiltrating CD8^+^ T cells 17. Our current data shows that FcγRIIB regulates the immunosuppressive activity of MDSCs, and blockade of FcγRIIB promotes the anti-tumor T cell response through inhibiting the accumulation and the immunosuppression role of MDSCs. Moreover, FcγRIIB signaling promotes apoptosis in mature B cells in the absence of BCR ligation 15. A recent study also demonstrates that FcγRIIB signaling promotes apoptosis in CD8^+^ T cells in a Fgl2-dependent manner 26. Here we found that elevated FcγRIIB expression on MDSCs did not promote apoptosis of MDSCs in tumor tissues, while the proportion of tumor-infiltrating gMDSCs increased. Mechanistically, tumor-derived GM-CSF increased the expression of FcγRIIB and subsequently promoted the differentiation of HPCs into GMPs and gMDSCs. Since tumor progression is associated with the abnormal differentiation of HPCs into MDSCs, and the gMDSC subset is the predominant MDSC population in tumor-bearing mice 38; but the underlying mechanism driving abnormal myeloid cell differentiation in cancer remains poorly understood. The bone marrow chimerism experiment demonstrated the critical function of FcγRIIB in regulating the immunosuppressive activity and differentiation of MDSCs. Our study did not exclude the contribution of other immune cells in the anti-tumor immunity of FcγRIIB KO mice, but outlines the critical role of FcγRIIB in promoting gMDSCs generation during tumor progression.

Stat3 signaling pathway is crucial for MDSC population expansion, and the activity of Stat3 increases in MDSCs during tumor-bearing conditions 39. In our present study, Stat3 signaling pathway was significantly suppressed in FcγRIIB-deficient HPCs than in WT HPCs, suggesting that FcγRIIB might regulate differentiation of HPCs *via* the Stat3 signaling pathway. Moreover, FcγRIIB-deficient HPCs were positively enriched in HALLMARK gene sets for development or differentiation of erythrocytes. Erythrocytes are normally developed from MEPs in BM, although chronic infections and malignancies also induce erythropoiesis 20. Tumor progression is often associated with anemia in patients 40. However, how tumor development influences medullar erythropoiesis remains poorly understood. The results of the present study showed that tumor development prevented the differentiation of HPC into MEPs, while FcγRIIB deficiency enhanced HPCs differentiation into MEPs, even in tumor-free mice. Interestingly, FcγRIIB-deficient HPCs showed decreased Csf2R expression and would be less sensitive to GM-CSF stimulation, suggesting an intrinsic role of FcγRIIB in suppressing medullar erythropoiesis during tumor-initiated anemia.

GM-CSF is an immune-modulatory cytokine that can promote the differentiation of immature progenitors into macrophages and DCs 41. This cytokine has been used as an immunostimulatory adjuvant to induce anti-tumor immunity 42. Nevertheless, it has been previously reported that GM-CSF production by tumors can directly suppress CD8^+^ T cell activity by upregulating MDSCs 43. Recent studies showed that knockdown of GM-CSF in tumor cells or blockade of GM-CSF with antibody reduces expansion of MDSCs, delays tumor progression, and enhances the efficiency of PD-L1 antibody 44, 45, while the underlying mechanism remains vague. The present study showed that GM-CSF promoted the expression of FcγRIIB on MDSCs by activating Sp1 signaling. Blockade of Sp1 signaling suppressed the expression of FcγRIIB and dampened the expansion of MDSCs in the TME. Aberrant expression or activation of Sp1 has been found in various types of cancers 46, 47, and increasing evidence suggests a crosstalk between Sp1 and Stat3 in tumors 48. Therefore, Sp1 and Stat3 may cooperatively activate targeted genes and mediate the immunosuppressive activity of MDSCs. Further investigation of regulatory pathways mediated by GM-CSF is necessary to expand the understanding of its role in tumor immunity.

In summary, we report that FcγRIIB contributes to the immunosuppressive activity of MDSCs and the differentiation of HPCs into gMDSCs under tumor conditions. Tumor cell-derived GM-CSF promotes Sp1 binding to the* FCGR2B* promoter to increase the expression of FcγRIIB that subsequently activates the Stat3 signaling pathway to promote generation of gMDSCs in the TME. Moreover, blocking FcγRIIB decreases MDSC infiltration, promotes CD8^+^ T cell activity in tumor-bearing mice, and improves the therapeutic efficacy of gemcitabine. These findings indicate that FcγRIIB is a potential anti-cancer target for immunotherapy.

## Materials and methods

### Human samples and databases

Peripheral blood was collected from healthy adult volunteers and patients with CRC in Chongqing University Cancer Hospital, Chongqing, China. All experiments involving human subjects were conducted in accordance with local, national, and international regulations and were approved by the Ethics Committee of the Chongqing University Cancer Hospital, Chongqing, China. All patients provided written informed consent in accordance to the declaration of Helsinki before enrolling in the study. Mononuclear cells in peripheral blood were freshly isolated over lymphocyte separation medium (0850494X, MP Biomedicals). The expression of FcγRIIb in normal colon, colon adenocarcinoma, colon mucinous adenocarcinoma and rectal adenocarcinoma tissues were determined through analysis of Kurashina Colon cancer datasets, which are available at Oncomine (http://www.oncomine.org/). All available TCGA data on Colon adenocarcinoma were obtained from the TCGA data portal (TCGA group). The RNA-seq data of colon cancer Patients (n = 597) were retrieved from cbioportal (http://www.cbioportal.org).

### Animals

C57BL/6 mice were obtained from the Animal Institute of the Academy of Medical Science (Beijing, China). C57BL/6 wild type (WT) mice were purchased from the Chinese Academy of Medical Sciences (Beijing, China). EM:06078 Fcgr2b Fcgr2bB6null B6(Cg)-Fcgr2btm12Sjv/Cnbc (FcγRIIb^-/-^ mice) and Fgl2^-/-^ mice were kindly provided by J.S. Verbeek (Leiden University Medical Center, The Netherlands) and S. Smiley (The Trudeau Institute, NY, USA), respectively. OT-1 transgenic mice were purchased from the Jackson Laboratory. These mice were backcrossed for at least nine generations onto a C57BL/6 background, and their homozygous wild type littermates were used as controls. All mice were kept in specific pathogen-free conditions under a 12 h light cycle, and were given a regular chow diet at Chongqing University Cancer Hospital. All animal experiments were approved by the ethics committee of the Army Medical University. All animal studies were conducted in accordance with the national and international Guidelines for the Care and Use of Laboratory, and with the approval of the Animal Care and Use Committee (IACUC) of Army Medical University and Chongqing University Cancer Hospital (Chongqing, China, AMUWEC2017294) and complied with the Declaration of Helsinski.

### Cell lines and treatment

Murine colon adenocarcinoma cell line MC38 and melanoma cell line B16F10 were purchased from Type Culture Collection of the Chinese Academy of Sciences (Shanghai, China) in 2015 and cultured in DMEM (HyClone) containing 10% FBS (Gibco) containing 10% FBS and 1% penicillin-streptomycin. Human monocyte THP-1 Cells were purchased from ATCC. Cells were routinely verified Mycoplasma-free using MycAwayTM-Color One-Step Mycoplasma Detection Kit (Yeasen Bio-technol) and the most recent date of testing was April 12, 2021. These cells were authenticated and certified by ChengDu Nuohe Biotech co., LTD (Sichuan, China).

For primary cell culture, single-cell suspensions of bone marrow (BM) cells derived from 8-10 week-old WT or KO mice were stained by anti-mouse Gr-1 particles (Cat No. 558111, BD Biosciences) and separated using the BD IMag Cell Separation Magnet. These harvested Cells were cultured in RPMI1640 medium containing 10% FBS supplemented with 20 ng/mL GM-CSF (315-03, PeproTech) and IL-6 (216-16, PeproTech) in the present or absence of STAT3-IN-1 (1μM, Cat No. S0818, Selleck) for 48 h to obtain BM derived MDSCs. For BM differentiation assays, BM cells from 8-10 week-old WT mice were cultured in RPMI1640 medium containing 10% FBS supplemented with 20 ng/mL GM-CSF (315-03, PeproTech) and IL-6 (216-16, PeproTech) in the present of Serum Amyloid protein/SAP (20 ng/mL, Cat No. ab276767, Abcam), C Reactive Protein (CRP) (20 ng/mL, Cat No. ab276840, Abcam) and Mouse IgG for 72 h. Mouse IgG was incubated with the goat F(ab′)_2_ anti-mouse IgG (Cat No. ab98659, Abcam) for 60 min in PBS at 37 °C before adding to cells.

### Tumor growth and treatments

To establish an *in vivo* tumor model, 1× 10^6^ MC38 or 5×10^5^ B16F10 cells were injected subcutaneously into the right flank of C57BL/6, FcγRIIb^-/-^ or Fgl2^-/-^ mice. Tumor growth was measured using calipers every 3 days. Tumor volume was calculated as follows: V= (length × width^2^) × 0.5. For Gr-1^+^cell depletion studies, 250 µg anti-Gr-1 neutralizing antibody (Cat No. BE0075, BioXcell, West Lebanon, USA) was administered *i.p.* every 3 days starting once tumors became palpable until the mice were sacrificed. FcγRIIb depletion in MC38-bearing mice was achieved by *i.p.* injections of 250 µg anti-mouse FcγRIIb antibody (clone AT128) every other day 49. For *in vivo* treatments of gemcitabine (GEM, 50 mg/kg, Cat No. S1149, Selleck) and Mithramycin A (Mith, 0.2 mg/kg, Cat No. HY-A0122, Med Chem Express), treatment was initiated once tumor volume was approximately 100 mm^3^, compounds were diluted in PBS and given *i.p.* every 3 days until the mice were sacrificed.

### Flow Cytometry (FCM)

Single-cell suspension samples of tumor, spleen and BM were harvested from euthanized mice at the indicated time points were prepared and blocked with rat IgG (10 μg/mL; Sigma) for at least 20 min on ice. Then, cells were labeled with the indicated antibodies (1:100) for 30 min at 4 °C. Dead cells were excluded by using a Fixable Viability Dye Efluor 780 (Cat No. 65-0865-14, eBioscience) following manufacturer's instructions. The panel of antibodies used in these experiments included CD11b (Cat No. 101208), Gr-1 (Cat No. 108426), CD33 (catalog no. 303414), HLA-DR (catalog no. 307610), CD3 (Cat No. 100206), CD4 (Cat No. 100412), CD8α (Cat No. 100706), CD11c (Cat No. 117308), CD103 (Cat No. 121414), Ly6G (Cat No. 127626), Ly6C (Cat No. 128008), F4/80 (Cat No. 123116), B220 (Cat No. 152406), PD-L1 (Cat No. 124308), CD45.1 (Cat No. 110708), CD45.2 (Cat No. 109814), Sca-1 (Cat No. 108106), c-kit (Cat No. 105808), CD16/32 (Cat No. 101331), CD34 (Cat No. 119310) and APC Annexin V Apoptosis Detection Kit with 7-AAD (Cat No. 640930) from Biolegend (San Diego, CA). FcγRIIb (Cat No. 17-0321-80 and PA5-47122) was purchased from thermofisher, Lineage (Cat No. 561317) was from BD. For staining of Foxp3 (Cat No. 14-5773-82, eBioscience), ARG1 (Cat No. 42284, GeneTex), iNOS (Cat No. MA5-17139, Thermo), IFN-γ (Cat No. 505810, Biolegend), granzyme B (GzmB; Cat No. 515403, Biolegend), Ki67 (Cat No. 12075, CST), S100A8 (Cat No. 50-9745-42, eBioscience), Stat3 (1:1000; Cat No. 9139, CST) and Sp1 (Cat No. ab227383, Abcam), cells were stained surface markers, then fixed and permeabilized with Foxp3/Transcription factor staining kit (Cat No. 00-5523-00, eBioscience), followed by primary/secondary antibodies staining according to the manufacturers' protocols. The proliferation and functional assay of CD8^+^ T cells were performed as previously described 10. CFSE probe was obtained from Dojindo (Cat No. C309). DCFH-DA (Cat No. S0033) probes were from Beyotime (Shanghai, China). FCM was performed on BD FACS Canto II platforms and results were analyzed with FlowJo software version 10.0.7 (TreeStar). The Mean fluorescence intensity (MFI) of antigen was determined by measuring the difference in mean channel fluorescence of positive staining cells subtract to unstained sample controls. Cell sorting was performed on an BD FACSAria II instrument (BD Biosciences).

### BM reconstitution assay

6-8-week-old male or female mice (CD45.1) were irradiated with 9.5 Gy (MultiRad 225, Faxitron). Subsequently, 1×10^7^ BM cells (resuspended in100μL PBS) from WT or FcγRIIb-KO mice (CD45.2) were injected intravenously *via* the tail vein. 2 weeks after BM transfer, mice were given antibiotic water (containing 10mg/mL Trimethoprim and Sulfamethoxazole). To confirm BM reconstitution, the peripheral blood MDSCs from chimeric mice were assessed by flow cytometry using antibody against CD45.1 (Cat No. 110708, Biolegend) and CD45.2 (Cat No. 109814, Biolegend). The tumor model experiments were initiated at 6 weeks after BM reconstitution.

### Transfection of shRNAs in MDSCs

Three pLKD-CMV-EGFP-2A lentivectors containing shRNAs targeting Sp1 or a lentivector containing scrambled shRNA were obtained from Genechem (Shanghai, China). Sorted mouse CD11b^+^Gr1^+^ cells from BM were plated at 1× 10^6^ cells per mL in 12-well plates and transduced with lentiviral particles (at MOI of 100) with 5µg/mL Polybrene (GenePharma). Cells were harvested and used for further experiments 3 days after transfection.

### ELISA

The GM-CSF expression in serum, BM and tumor tissues of MC38 tumors bearing WT and FcγRIIb^-/-^ mice were measured using a Mouse GM-CSF ELISA Kit (Cat No. 432207, Biolegend) according to the manufacturer's instruction.

### Immunofluorescence

Mice tumor samples were fixed with 4% formaldehyde for 15 min and tissue sections were then incubated in 10% normal goat serum for 1 h. The cells were then incubated with the primary antibody Gr-1 (1:50, Cat No. MAB1037, R&D Systems) and CD8α (1:50, Cat No. GTX74642, GeneTex) at 4 °C overnight. The secondary antibody Anti-rat IgG (H+L) Alexa Fluor 488 Conjugate (Cat No. 4416, CST) was used at a 1:200 dilution for 1 h. DAPI was used to stain the nucleus at a concentration of 100 ng/mL. Then, the sections were imaged on a Leica TCS SP5 confocal microscope (Leica Microsystems).

### Quantitative real-time PCR

Total RNA was extracted from cells using RNAiso Plus (TAKARA, Japan) and the RNA concentration in the samples was measured using NanoDrop 2000 (Thermo Scientific). 1 µg total RNA was converted to complementary DNA (cDNA) using the PrimeScript RT-PCR Kit (RR014A, Takara) according to the manufacturers' instructions. Quantitative real-time PCR (qPCR) was performed using TB Green Fast qPCR Mix Kit (RR430A, Takara) on a CFX384 system (BIO-RAD), and the relative quantification (2-^ΔΔCt^) method was used to analyze gene expression. β-actin mRNA was used as a reference for mRNA quantification. All qPCR experiments were repeated at least three times. Primer sequences were as following:

*Cxcl1*-F: (5′- GCCTCTAACCAGTTCCAGCA-3′); *Cxcl1*-R: (5′-TTGAGGTGAATCCCAGCCAT-3′); *Cxcl2*-F: (5′-GGCAAGGCTAACTGACCTGG-3′); *Cxcl2*-R: (5′-CTCAGACAGCGAGGCACATC-3′); *Cxcl5*-F: (5′-CTCTGTTCAGCTATTGGACGC-3′); *Cxcl5*-R: (5′-GAACACTGGCCGTTCTTTCC-3′); *Cxcl12*-F: (5′-GGTGCTCAAACCTGACGGTA-3′); *Cxcl12*-R: (5′-GGCAGCTCCTCTTTGGCTTA-3′).

### RNA sequencing library construction

Total RNA was isolated from sorted BM HPCs (lin^-^, Sca-1^-^, c-kit^+^) from WT and FcγRIIb^-/-^ tumor-bearing mice on day 21 after MC38 tumor cell implantation. The RNA-seq library for these RNA samples was constructed according to the strand-specific RNA sequencing library preparation protocol. mRNA transcripts were enriched by two rounds of poly-(A+) selection with Dynabeads oligo-(dT) 25 (Invitrogen) before library construction. The prepared libraries were sequenced on an Illumina Novaseq 6000 platform.

### Chromatin immunoprecipitation (ChIP) assay

Chromatin immunoprecipitation (ChIP) assays were performed using a Simple ChIP Plus Enzymatic Chromatin IP Kit (Cat No. 9005, CST) according to the manufacturers' instructions. In brief, chromatin from BM-derived MDSCs were chemically cross-linked with 1% formaldehyde solution for 10 min at room temperature and quenched with 0.125 M glycine. The fixed cells were resuspended, and lysised DNA to length of approximately 150-900 bp and immunoprecipitated with ChIP-validated Sp1 antibody (Cat No. ab227383, Abcam) and IgG (Cat No. 3900, CST). DNA samples were purified and amplified by four pairs of ChIP-PCR primers that designed to amplify the region containing each Sp1 binding site at the FcγRIIb promoter. The amplified products were analyzed by agarose gel electrophoresis. The primer sequences used were as following:

-1385~-1376-F ACCTCTACTGCCATCAGC,

-1385~-1376-R TTCCACCCTATCTACCCT;

-562~-553-F CGAATGGGTAGAAATGTTGATC,

-562~-553-R GTCTGTGCCCTAGTCCTGAA.

### FcγRIIB promoter luciferase reporter assay

To measure the effect of Sp1 on FcγRIIB expression in HPCs, the FcγRIIB promoter region (-1500 ~ +1 from the transcription starting site) was synthesized by GenScript co., LTD (Nanjing, China) and subcloned into pGL3-basic vector (Promega, Madison, WI, USA), Sp1 binding site 5'- GGGGCGGGGC was mutated to 5'-AAGGCAAGGC. Lentivectors containing Sp1 overexpression or a lentivector containing mock were obtained from Genechem (Shanghai, China). The HPCs were transfected with 1 μg of reporter vector and 20 ng of pSV-Renilla expression vector (mock and Sp1) using Advanced DNA RNA Transfection Reagent (Cat No. AD600025, Zeta-life). Transfected cells were incubated for 48 h, Luciferase and Renilla activities were measured using the dual-luciferase reporter system kit (Cat No. E1910, Promega). The transfection was performed according to the manufacturer's protocol.

### Western blotting

Single-cell suspensions of BM cells from 8-10 week-old WT or KO mice were stained by anti-mouse Gr-1 particles (Cat No. 558111, BD Biosciences) and separated by the BD IMag Cell Separation Magnet. These harvested cells were cultured in RPMI1640 medium containing 5% FBS supplemented with 10% tumor supernatants from MC38 cells or 20 ng/mL GM-CSF for 48 h. Cells were lysed by RIPA lysis buffer, and the cell lysates were incubated on ice for 30 min and centrifuged at 13,000g, 4 °C for 15 min before the supernatants were collected. Western blot analysis was performed as previously described 10. The primary antibodies included anti-Sp1 (1:1,000; Cat No. ab227383, Abcam) and anti-Actin (1:1,000; Cat No. A1978, Sigma-Aldrich).

### Statistical analysis

All results were confirmed in at least three independent experiments and were expressed as means ± SD. Mann-Whitney test and one- or two-way ANOVA were used for calculation statistical significance with GraphPad Prism software (version 8.0). The Kaplan-Meier method was used to assess overall survival, and the differences in survival curves were analyzed using the log-rank test. *P* < 0.05 was considered statistically significant.

## Supplementary Material

Supplementary figures.

## Abbreviations

BM: Bone marrow
ChIP: Chromatin immunoprecipitation
CMPs: Common myeloid progenitors
CRC: Colorectal cancer
CRP: C Reactive Protein
CTLs: Cytotoxic T lymphocytes
DCs: Dendritic cells
FCM: Flow Cytometry
FcγRIIB: Fc gamma receptor IIB
Fg12: Fibrinogen-like protein 2
GM-CSF: Granulocyte-macrophage colony stimulating factor
GMP: Granulocyte/macrophage progenitors
GSEA: Gene set enrichment analysis
HPCs: Hematopoietic progenitor cells
Igs: Immunoglobulins
iNOS: Inducible nitric oxide synthase
ITIM: Immunoreceptor tyrosine-based inhibitory motif
MDSCs: Myeloid-derived suppressor cells
MEP: Megakaryocyte/erythrocyte progenitors
Mith: Mithramycin A
PD-L1: Programmed death-ligand 1
PGE2: Prostaglandin E2
qPCR: Quantitative real-time PCR
RNA-seq: RNA sequencing
ROS: Reactive oxygen species
SAP: Serum Amyloid protein
Sp1: Specificity protein 1
TAMs: Tumor-associated macrophages
TME: Tumor microenvironment
TGFβ: Transforming growth factor-β
Tregs: Regulatory T cells
WT: Wild type
