# Supplementary material for: FcγRIIB potentiates differentiation of myeloid-derived suppressor cells to mediate tumor immunoescape
Source: Theranostics. 2022 Jan 1;12(2):842–58. doi: 10.7150/thno.66575 (PMC8692894; doi:10.7150/thno.66575)
Supplement: Supplementary file 1 — Supplementary figures. [file thnov12p0842s1.pdf]

Supplementary information

Supplementary Figures

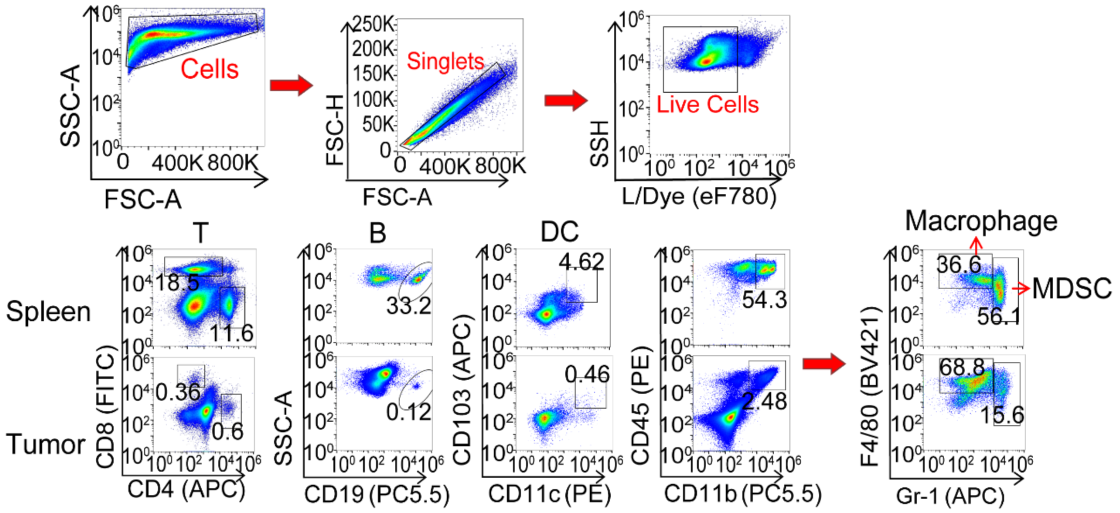

Figure S1. Gating strategy of immune cells in MC38 tumor bearing mice. Gating strategy for T cells ($CD3^+CD8^+$ or $CD3^+CD4^+$), B cells ($CD19^+$), DCs ($CD11c^+CD103^+$), MDSCs ($CD11b^+CD45^+Gr1^+$) and macrophages ($CD11b^+CD45^+F4/80^+$) in mouse spleen and tumor.

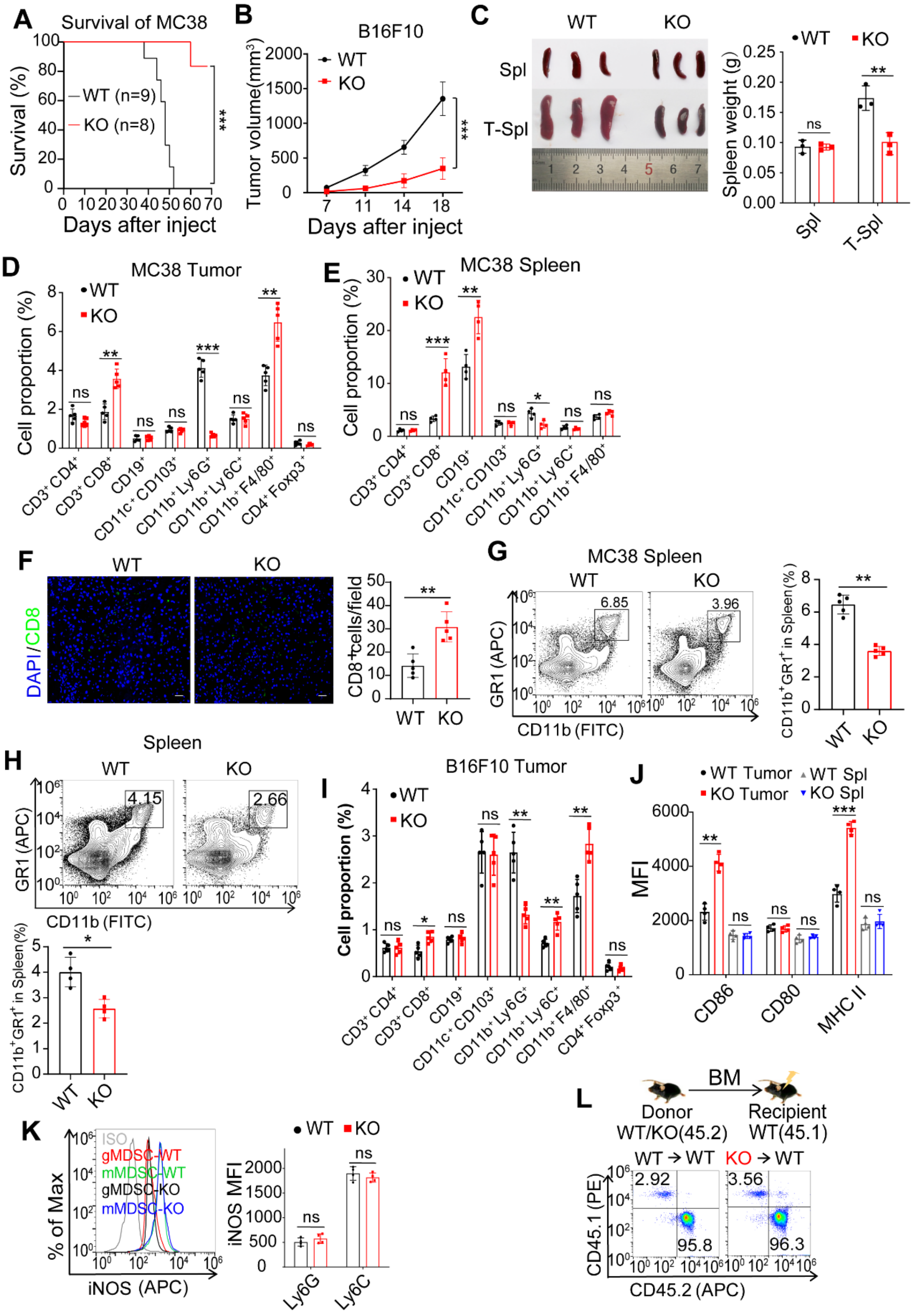

Figure S2. FcγRIIB deficiency reduces MDSCs in the TME. (A) Survival of MC38 tumor-bearing WT or FcγRIIB$^{-/-}$ (KO) mice, one of three representative experiments is shown, with n = 8-9 mice each group (log-rank [Mantel-Cox] test, $p < 0.001$). **(B)** A total of 5×10^5 B16F10 melanoma cells were subcutaneously injected into WT or KO mice, tumor growth was monitored every 3 or 4 d. **(C)** WT or KO mice were injected

subcutaneously with 10^6 MC38 cells, mice were sacrificed at day 21 post-grafting, and spleen from tumor free mice (Spl) and tumor bearing mice (T-Spl) were collected and weighed, representative spleen images are shown, $n = 3$. **(D** and **E)** WT or KO mice were injected subcutaneously with 10^6 MC38 cells, Mice were euthanized on day 14, the frequency of T cells, B cells, DCs, MDSCs, macrophages and Tregs in tumor tissues or spleen were determined by FCM. **(F)** The frequency of $CD11b^+Gr1^+$ MDSCs in spleen from WT or KO tumor bearing mice was assessed 14 days after MC38 tumor inoculation, $n = 5$. **(F)** The $CD8^+$ cells in MC38 tumor tissue sections from WT or KO mice was detected by immunofluorescence. Scale bars: 50 µm. **(G)** The frequency of $CD11b^+Gr1^+$ MDSCs in spleen from WT or KO tumor bearing mice was assessed 14 days after MC38 tumor inoculation, $n = 5$. **(H)** The frequency of $CD11b^+Gr1^+$ cells in spleen from WT or KO naïve mice was determined by FCM, $n = 4$. **(I)** WT or KO mice were injected subcutaneously with 5×10^5 B16F10 melanoma cells for 14 d, the frequency of T cells, B cells, DCs, MDSCs, macrophages and Tregs in tumor tissues were determined by FCM, $n = 4$. **(J)** CD86, CD80 and MHC II expression for $CD11b^+F4/80^+$ cells from tumor and spleen were determined by FCM, $n = 4$. **(K)** iNOS expression in gMDSCs and mMDSCs from WT or KO MC38 tumor tissues were analyzed by FCM; $n = 4$. (**L)** Recipient mice were irradiated and then received WT or $Fc\gamma RIIb^{-/-}$ mouse-derived BM cells. CD45.1 and CD45.2 expressions on peripheral blood MDSCs were detected after BM reconstitution. Data are expressed as means ± SD. *$P < 0.05$, **$P < 0.01$, ***$P < 0.001$, by Mann-Whitney test. *ns,* no significant difference.

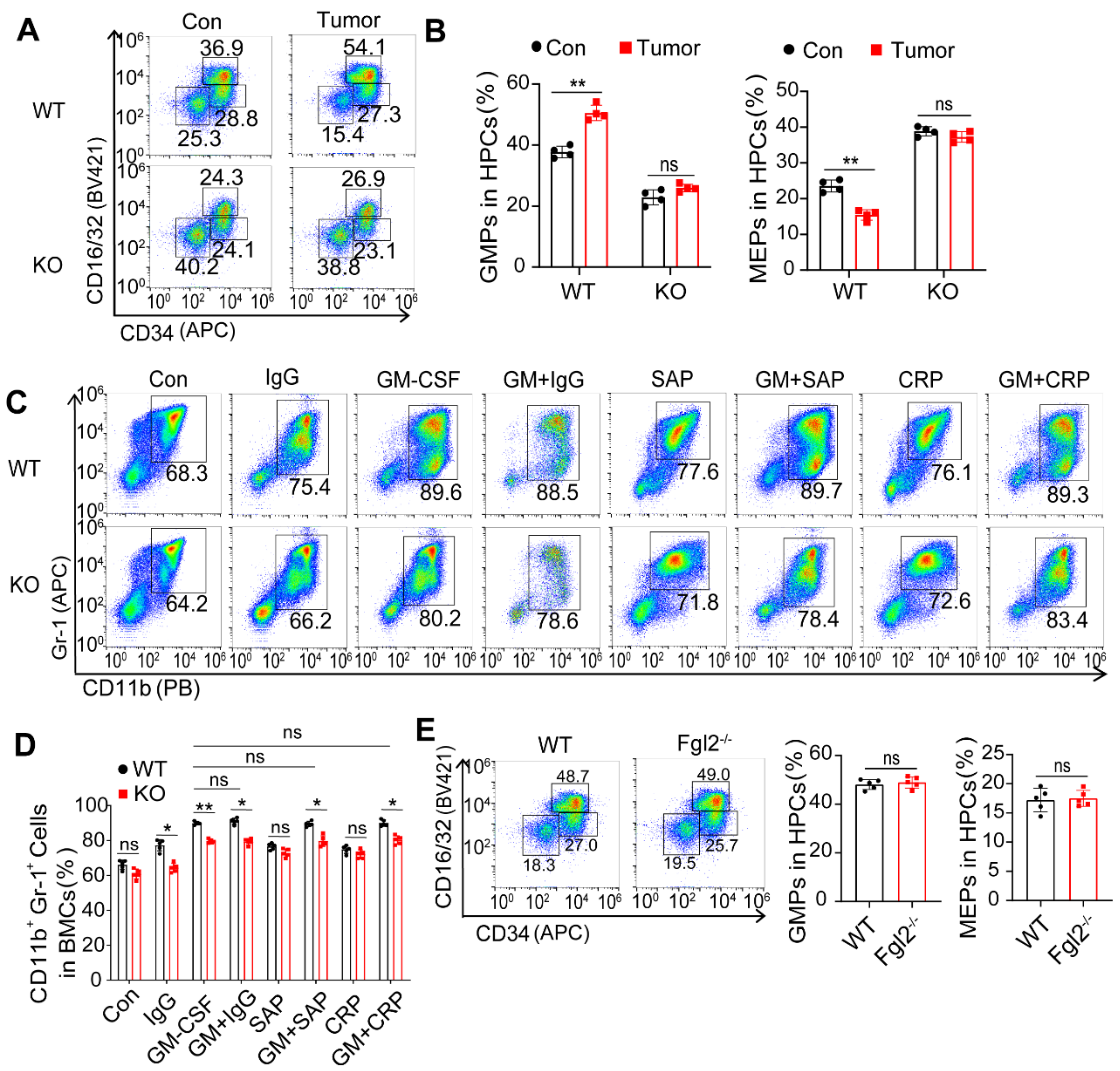

Figure S3. FcγRIIB promotes the differentiation of GMPs from HPCs independent of IgG, SAP, CRP and Fgl2. (A) The percentages of GMPs, CMPs and MEPs in HPCs from WT and KO tumor free or tumor bearing mice were analyzed**. (B)** Statistical results of **A**, *n* = 5). **(C)** WT and KO BMs were treated with GM-CSF/IL-6 (20 ng/mL) to induce MDSCs differentiation for 72h in the presence or absence of IgG (1 μg/mL), SAP (20 ng/mL) or CRP (20 ng/mL). Cells were then harvested and MDSCs were assessed *via* FCM. **(D)** Statistical results of (**C,** *n* = 4). **(E)** WT or Fgl2$^{-/-}$ mice were injected subcutaneously with 10^6 MC38 cells, mice were sacrificed at day 14 post-grafting of MC38 cells and the percentages of GMP, CMP and MEP in HPCs were analyzed, *n* = 5. Data are expressed as means ± SD. **P < 0.01, Mann-Whitney test. *ns,* no significant difference.

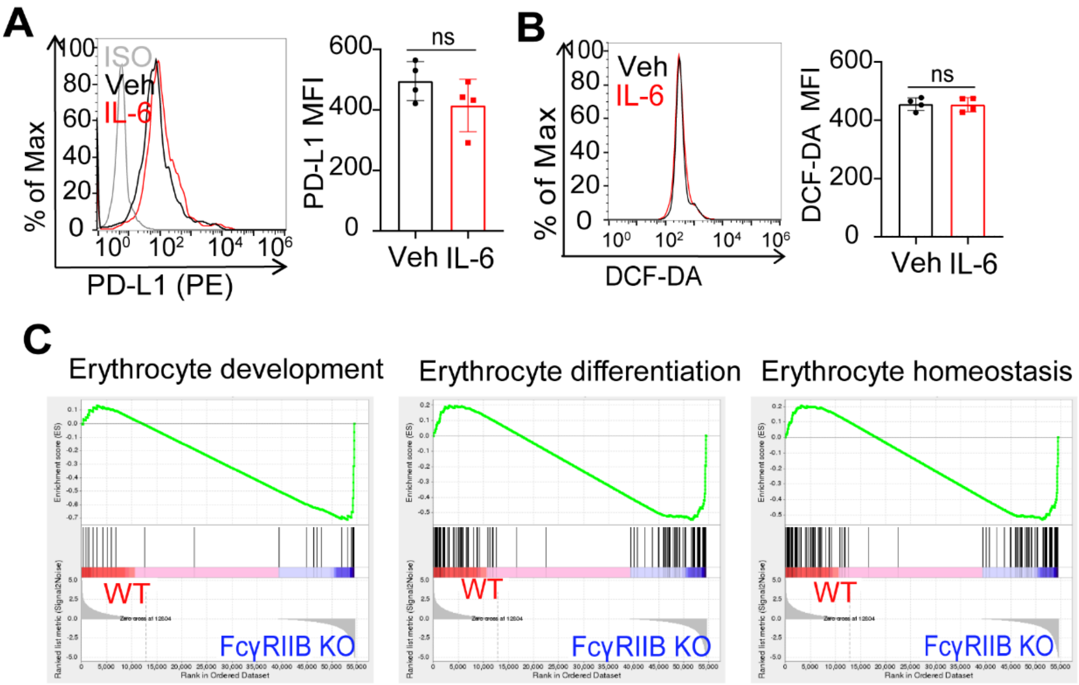

Figure S4. FcγRIIB deficiency promotes HPCs differentiation to MEPs through impairing Stat3 pathway. (A and B) Isolated Gr-1^{+} cells from KO mice were treated with IL-6 (20ng/mL) for 48 h, the expression of PD-L1**,** and DCF-DA were measured, n = 4. **(C)** Enrichment plot of the HALLMARK erythrocyte development, erythrocyte differentiation and erythrocyte homeostasis signaling pathway for the comparison between WT or FcγRIIB KO HPCs. Data are expressed as means ± SD. Mann-Whitney test. *ns,* no significant difference.

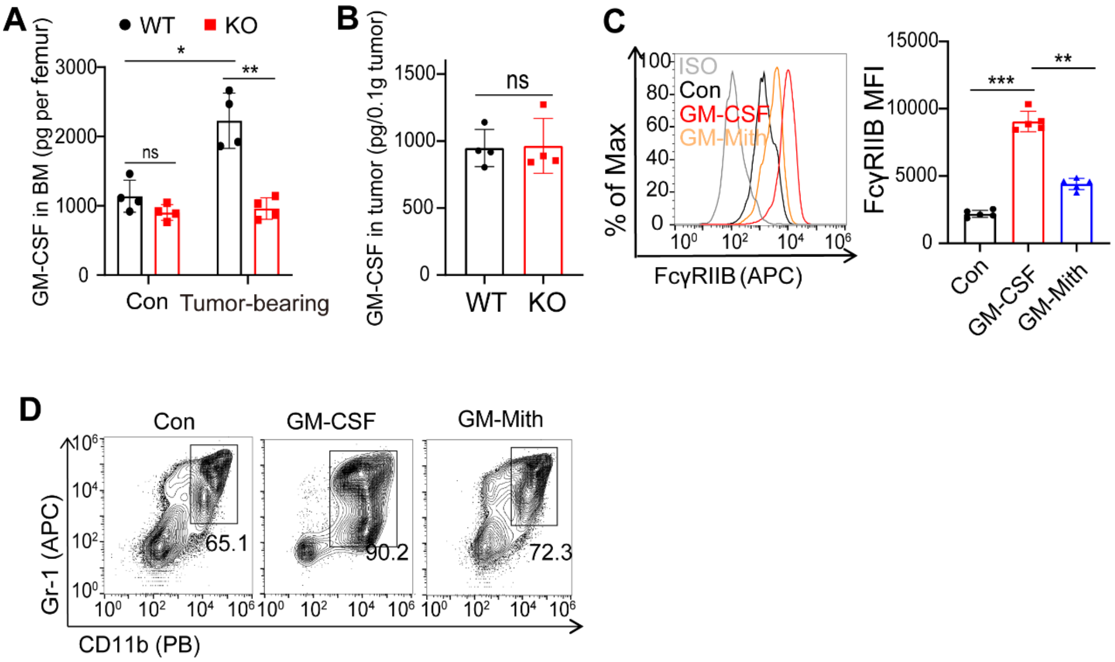

Figure S5. Tumor-bearing WT mice express high levels of GM-CSF in BM. (A and B) WT or KO mice were injected subcutaneously with 10^6 MC38 cells, mice were sacrificed at day 21 post-grafting, the GM-CSF expression levels in BM and tumor tissues (*n* = 4) were measured using a Mouse GM-CSF ELISA kit, *n* = 4. **(C)** THP1 cells were treated with GM-CSF (20ng/mL) for 48 h, in the presence or absence of Mith and FcγRIIB expression were assessed using FCM; *n* = 5. **(D)** BM cells were treated with GM-CSF in the presence or absence of 1 μl PBS (Con) or Mithramycin A (Mith, 20 nM) for 48 h, the percentages of MDSCs were assessed. Data are expressed as means ± SD. *P < 0.05, **P < 0.01, ****P* < 0.001, Mann-Whitney test. *ns,* no significant difference.

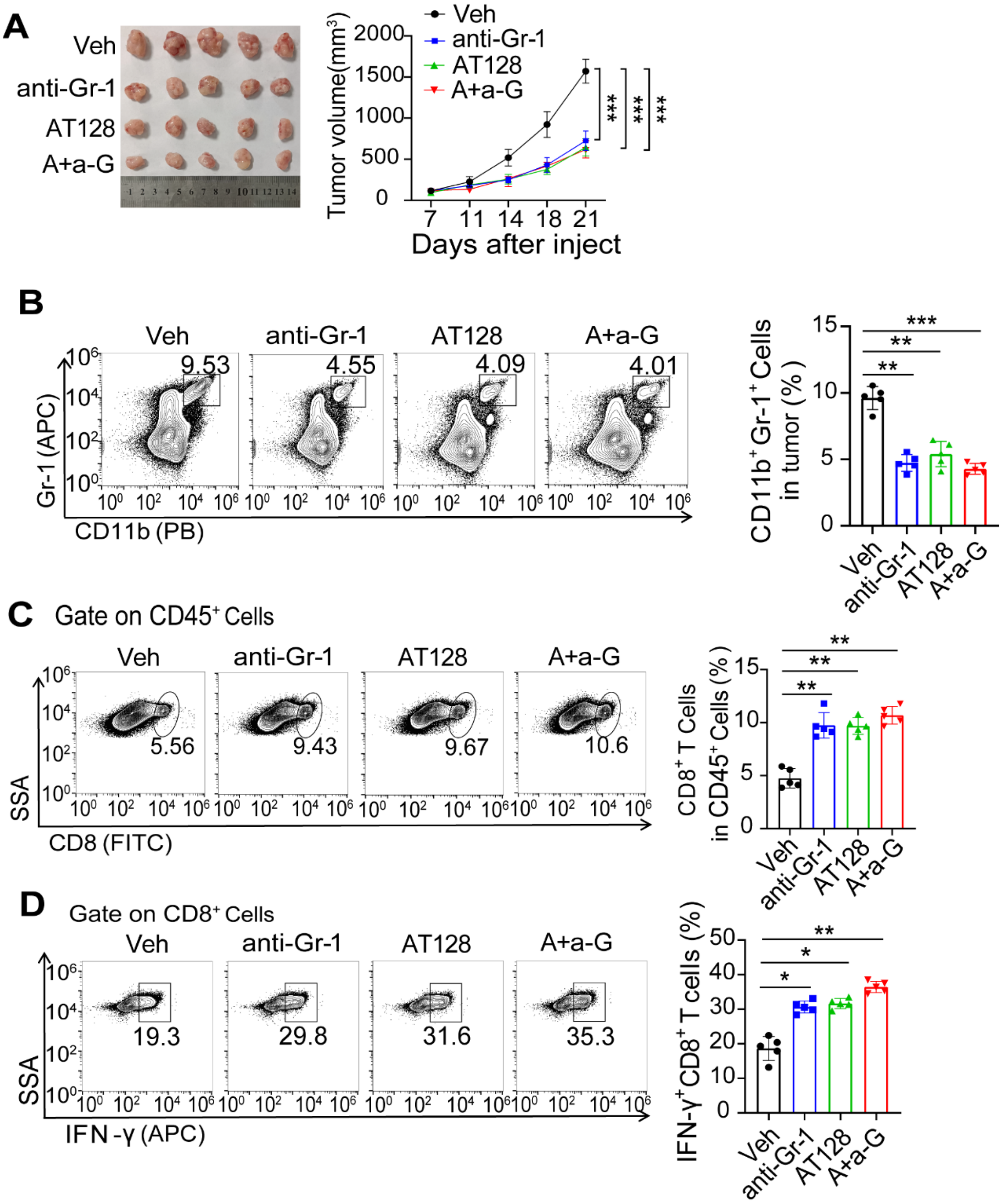

Figure S6. Blocking FcγRIIB signaling decreases MDSC infiltration and promotes CD8$^+$ T cell activity. (A-D) WT mice were injected subcutaneously with MC38 tumor cell. After 7 days, tumor-bearing mice were injected with PBS (Veh), 250 μg of the anti-mouse Gr-1 antibody, 250 μg anti-mouse FcγRIIb antibody (AT128) or combined treatment (A+a-G). Tumor growth was monitored for 21 days (**A**). All mice were euthanized on day 21 after tumor injection, the percentages of MDSCs, the percentages of CD8$^+$ T cells in tumor tissues (**B**), and the percentage of CD8$^+$ T cells (**C**) producing IFN-γ (**D,** n = 5) in tumor tissues were analyzed by FCM. One-way ANOVA with Tukey multiple comparison posttest was used to evaluate statistical significance. *P < 0.05, **P < 0.01, ***P < 0.001. *ns,* no significant difference.

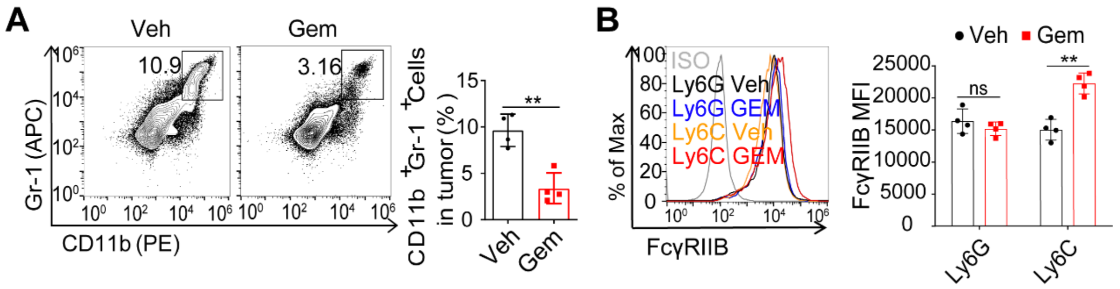

Figure S7. FcγRIIB is upregulated in tumor-infiltrating MDSCs from mice received gemcitabine treatment. (A) WT mice were injected subcutaneously with MC38 tumor cell. After 7 days, tumor-bearing mice were injected *i. p.* with 100μl PBS (Veh), gemcitabine (Gem, 50 mg/kg), the percentages of $CD11b^{+}Gr1^{+}$ in MC38 tumor tissues was analyzed 21 days after tumor inoculation, $n = 4$. **(B)** The expression of FcγRIIB on gMDSCs and mMDSCs from MC38 tumor were determined, $n = 4$. Data are expressed as means ± SD. **P < 0.01, by Mann-Whitney test. *ns,* no significant difference.
